# Supplementary material for: IM30 IDPs form a membrane-protective carpet upon super-complex disassembly
Source: Commun Biol. 2020 Oct 21;3:595. doi: 10.1038/s42003-020-01314-4 (PMC7577978; doi:10.1038/s42003-020-01314-4)
Supplement: Supplementary file 6 — Description of Additional Supplementary Files [file 42003_2020_1314_MOESM6_ESM.pdf]

Description of Additional Supplementary Files

File Name: Supplementary Data 1
Description: Data used to generate graphs and charts shown in Figs. 1a, 1b, 1d, 1e, 2a-f.

File Name: Supplementary Data 2
Description: HDX-MS data.
